# Quantitative Assessment of First Nations Drinking Water Distribution Systems for Detection and Prevalence of Thermophilic *Campylobacter* Species

**DOI:** 10.3390/ijerph191710466

**Published:** 2022-08-23

**Authors:** Izhar U. H. Khan, Anita Murdock, Maria Mahmud, Michel Cloutier, Thomas Benoit, Sabrin Bashar, Rakesh Patidar, Ruidong Mi, Bahram Daneshfar, Annemieke Farenhorst, Ayush Kumar

**Affiliations:** 1 Ottawa Research and Development Centre, Agriculture and Agri-Food Canada, 960 Carling Ave., Ottawa, ON K1A 0C6, Canada; 2 Department of Microbiology, University of Manitoba, Winnipeg, MB R3T 2N2, Canada; 3 Department of Biochemistry, Microbiology and Immunology, University of Ottawa, Ottawa, ON K1H 8M5, Canada; 4 Department of Soil Science, University of Manitoba, Winnipeg, MB R3T 2N2, Canada

**Keywords:** First Nations communities, households, tap water, *Campylobacter* spp.

## Abstract

Water is considered a major route for transmitting human-associated pathogens. Although microbial water quality indicators are used to test for the presence of waterborne pathogens in drinking water, the two are poorly correlated. The current study investigates the prevalence of thermophilic DNA markers specific for *Campylobacter* spp. (*C*. *jejuni* and *C. coli*) in source water and throughout the water distribution systems of two First Nations communities in Manitoba, Canada. A total of 220 water samples were collected from various points of the drinking water distribution system (DWDS) between 2016 and 2018. Target *Campylobacter* spp. were always (100%) detected in a home with a fiberglass (CF) cistern, as well as the community standpipe (SP). The target bacteria were also frequently detected in treated water at the Water Treatment Plant (WTP) (78%), homes with polyethylene (CP) (60%) and concrete (CC) (58%) cisterns, homes with piped (P) water (43%) and water truck (T) samples (20%), with a maximum concentration of 1.9 × 10^3^ cells 100 mL^−1^ (*C. jejuni*) and 5.6 × 10^5^ cells 100 mL^−1^ (*C. coli*). Similarly, target bacteria were detected in 68% of the source water samples with a maximum concentration of 4.9 × 10^3^ cells 100 mL^−1^ (*C. jejuni*) and 8.4 × 10^5^ cells 100 mL^−1^ (*C. coli*). Neither target *Campylobacter* spp. was significantly associated with free and total chlorine concentrations in water. The study results indicate that there is an immediate need to monitor *Campylobacter* spp. in small communities of Canada and, particularly, to improve the DWDS in First Nations communities to minimize the risk of *Campylobacter* infection from drinking water sources. Further research is warranted in improving/developing processes and technologies to eliminate microbial contaminants from water.

## 1. Introduction

Human-associated bacterial, protozoal and viral gastroenteritis-causing waterborne pathogens are responsible for drinking-water-related infectious disease outbreaks worldwide [1]. The contamination of drinking water production and distribution systems by microbial pathogens can pose significant health risks to the public. Drinking water intake sources are highly susceptible to fecal contamination from various origins that may contain pathogenic bacteria. Though the World Health Organization states in its 2004 recommendations [2] that the drinking water should be free from any microbial contaminant that can pose a risk to human health, appropriate treatment strategies remain lacking across the globe to remove harmful contaminants, pathogenic bacteria that may persist in drinking water distribution systems (DWDS) post-treatment.

Given that there is a high diversity of human-associated pathogenic bacteria, protozoa, and viruses that can be transmitted by contaminated water, it is difficult and costly to directly measure and regularly monitor all pathogens in the treated drinking water samples. Therefore, Canada and other countries have developed microbial drinking water quality standards and guidelines for routine monitoring of drinking water using indicators of fecal contamination, namely *Escherichia coli* and total coliforms [3]. According to these guidelines, a 100 mL drinking water sample should be free of fecal indicator organisms [2,4]. Although drinking water supplies in Canada are considered among the safest in the world, a significant number of First Nations communities remain under drinking water advisories due to microbial contamination [5,6,7]. As of March 2021, 58 long-term drinking water advisories persist in 38 First Nations communities across Canada [8]. However, previous studies have shown that fecal indicator bacteria can be common in drinking water, even in First Nation communities, even those with access to functioning water treatment plants [7,9,10]. This suggests that more rigorous water testing is needed at the household-level to better assess the urgency of improving safe drinking water supplies to First Nations families. This may require testing for pathogenic bacteria in addition to the commonly used fecal indicator bacteria.

Although in drinking-water-related *Campylobacter* outbreaks, *E. coli* or total coliforms were not always detected in the source or treated water samples, the possible reasons for the low detection rate could be due to a combination of too few samples and small sample volumes, samples were not continuously taken, or limited sampling points in the water distribution system [11,12,13]. Although *E. coli* or total coliforms are used as a predictor for the potential presence of pathogens in the water, a poor correlation between these microbial indicators and pathogens has been reported [14,15]. Therefore, expanding the monitoring of waterborne human pathogens in DWDS is critical to ensure the provision of safe drinking water to the communities.

Among several bacterial human-associated pathogens, *Campylobacter* has been identified as one of the major global causative agents of gastrointestinal infections and *Campylobacter jejuni* (*C. jejuni*) and *Campylobacter coli* (*C. coli*) are the leading cause of campylobacteriosis in humans [16]. These species can commonly occur in the gastrointestinal tracts of humans, bovines, pigs, and birds; therefore, municipal sewage discharges, agricultural runoff, animal or bird feces can contaminate surface water and groundwater that are used as a source water for water treatment [17,18,19]. The main transmission routes of infection in humans are generally via direct contact with companion animals and livestock, drinking contaminated water, consuming infected meat (e.g., undercooked poultry) and unpasteurized milk products [20,21]. In the past, *Campylobacter*-associated outbreaks in Canada have been directly linked to the contamination of surface water and groundwater from animals and animal waste on farms located near drinking water sources [22,23,24]. However, there have been no recent outbreaks, which is perhaps a testament to the effectiveness of water treatment systems in the majority of communities.

Culture-based methods have been used traditionally to monitor the microbial quality of water but these methods are likely to miss cells that are viable but go under a non-culturable state due to stress or injury and may not be cultured in the lab environment. Moreover, these methods cannot be applied in monitoring and investigating a large number of samples for rapid detection and identification of causative agents [25]. With recent advances in molecular technology that have led to enhanced sensitivity as well as user-friendly approaches, culture-independent nucleic-acid-based quantitative real-time PCR assay offers a rapid tool for detecting and measuring the total number of viable and non-viable cells of pathogenic and microbial water quality indicators in environmental and food sources [25,26,27,28].

In this study, we aimed to quantitatively assess the water quality of First Nations DWDS by investigating the prevalence and concentration of *C. jejuni* and *C. coli* in pre-(source) and post-treatment drinking water, as well as community lagoons and septic tanks, as both reference sites, and potential sources of contamination. We also investigated an assessment of these target species and their association with free and total chlorine and their inhibition and resistance in various sampling points.

## 2. Materials and Methods

### 2.1. Site Description and Water Sampling

Participating First Nations communities are located approx. 400 km northwest (Community B) and 450 km northeast (Community D) from the City of Winnipeg, the provincial capital of Manitoba, Canada. Both communities have conventional water treatment systems that utilize a combination of coagulation, flocculation, sedimentation, filtration, and disinfection with chlorine (sodium hypochlorite solution). Community B was additionally equipped with a reverse osmosis system and a dissolved air floatation system for clarification (sedimentation). In each community, only about one in every four households has access to piped water (directly connected to the water treatment plant, WTP). Three out of every four households in Community B rely on trucked water delivered to individual holding tanks (cisterns). These cisterns tend to be underground and constructed from either concrete or polyethylene materials.

**Figure 1:**
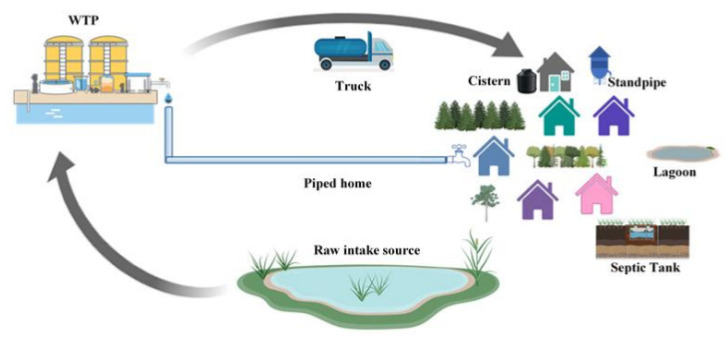
Schematic diagram showing various sampling points in DWDS of two First Nation communities investigated in this study.

For microbiological analysis, water samples were collected from various distribution systems, including pre-treatment (raw) intake (source) water taken at a tap in the water treatment plant (S), post-treatment water from the water treatment plant (WTP), community standpipe (SP) (only for Community D), water delivery trucks (T), taps from homes directly piped from WTP (P) or connected with one of cistern (polyethylene (CP), concrete (CC), fiberglass (CF)) types. The detailed descriptions and illustrations of sampling sites are given in Table 1 and Figure 1. In addition, samples from the community lagoon (L) and septic tanks (ST) were also collected from Community B to identify the potential sources of fecal contamination and assess the prevalence of *Campylobacter* spp.

In an initial sampling regimen, two sampling events were carried out in Community B between June and August 2017, followed by four subsequent samplings between April and October 2018 that were carried out in Community B. For a comparative analysis of two DWDS, water sampling in Community D was conducted in October 2018. Water sampling was carried out according to the standard SM 9060A and SM 9060B methods for sample bottle pre-treatment and sample preservation and storage as previously described [29]. Free residual and total chlorine were measured at the time of sampling using a Hatch Chlorine Pocket Colorimeter II (VWR, Mississauga, ON, Canada), following the manufacturer’s instructions according to USEPA DPD Method 8021 (Hach Company, Ames, IA, USA, 2002). Water samples were kept in a cooler on ice and stored at 4 °C overnight and shipped to the University of Manitoba laboratory. Analysis was performed immediately upon receiving samples.

### 2.2. Nucleic Acid Extraction

For direct DNA-based quantitative detection and identification of *C. jejuni* and *C. coli*, 200–700 mL (based on the turbidity of the sample) of drinking/source water and 10–50 mL of sewage water samples were initially filtered through sterile polyethersulfone membranes (0.22 µm pore size; 47 mm diameter; Pall Corporation, Mississauga, ON, Canada). The total genomic DNA was extracted using DNeasy PowerWater (QIAGEN, Germantown, MD, USA) DNA extraction kit following the manufacturer’s protocol. In addition, a sterile filter membrane was used as a negative control. The DNA quality, concentration and purity were assessed using a NanoDrop 2000 spectrophotometer (Thermo Scientific, Waltham, MA, USA) and Qubit 3.0 fluorometer (Thermo Fisher Scientific, Waltham, MA, USA). The DNA (concentration ranged from 0.5 to 238.5 ng µL^−1^) was stored at −20 °C for further quantitative real-time PCR assays.

### 2.3. Real-Time PCR Assays for Quantitative Detection of C. jejuni and C. coli

To quantify the total number of cells (per 100 mL) of each target species in the sampled water, standard curves were initially developed using the *C. jejuni* ATCC 29428 and *C. coli* ATCC BAA-971 reference strains. For the standard curve, the cells of each reference species were grown in Bolton broth containing antibiotic supplement under microaerophilic condition (5% O_2_, 10% CO_2_, and 85% N_2_) at 42 °C for 48 h. In order to quantify culture suspensions within a target range (10^8^ cell mL^−1^), the cell concentration (colony forming units (CFU) mL^−1^)) was measured by spread plating on Modified Karmali Agar containing antibiotic supplement under the same incubation condition. For assessing the purity and sensitivity of the assay, the known number of cells were spiked in autoclaved sewage water and filtered through a 0.22 μm sterile nitrocellulose filter. In addition, for sterility testing, autoclaved water was also filtered and used as a control. DNA was extracted from the filter using the DNeasy PowerWater (QIAGEN) DNA extraction kit per the manufacturer’s instructions. The extracted DNA of each species was serially diluted, corresponding to a decreasing concentration of cells (from 10^8^ to 10^1^ cells mL^−1^) for each target species. For generating a standard curve, each real-time quantitative PCR (qPCR) assay reaction was run in triplicate, and the crossing point (Cp) value for each set of reactions was plotted against each DNA concentration equivalent to the cell number as previously described [28].

For detection and quantitation of a total number of *C. jejuni* cells 100 mL^−1^, 20 μL TaqMan reaction mixture containing 10–40 ng μL^−1^ concentration of DNA template, 10 μL of Bio-Rad SsoAdvanced™ Universal Probes Supermix (BioRad, Hercules, CA, USA), and 0.5 μM of each *C. jejuni*-specific *hip*O gene-based forward (5′-CTG CTT CTT TAC TTG TTG TGG CTT T -3′), reverse (5′- GCT CCT ATG CTT ACA ACT GCT GAA T -3′) primers, and 0.04 μM of probe (FAM- CAT TGC GAG ATA CTA TGC TTT G -IABkFQ) [30]. The amplification reaction was carried out with an initial denaturation at 95 °C for 2 min followed by 50 cycles consisting of denaturation at 95 °C for 10 s, annealing at 60 °C for 20 s, and elongation at 72 °C for 30 s.

However, for *C. coli*, an SYBR Green-based qPCR assay was performed with a 20 μL reaction mixture containing DNA template (10–40 ng μL^−1^), 10 μL of Bio-Rad SsoAdvanced™ SYBR^®^ Green Supermix (Bio-Rad, Hercules, CA, USA), 0.5 μM of 16S-23S rDNA internal transcribed spacer (ITS) region-based forward (5′- GAA GTA TCA ATC TTA AAA AGA TAA -3′) and reverse (5′- AAA TAT ATA CTT GCT TTA GAT T -3′) primers [31] and 0.012 μg μL^−1^ of bovine serum albumin. The amplification reaction was initiated with a denaturation step at 98 °C for 3 min, followed by 50 cycles of repeating amplification of denaturation at 98 °C for 10 s, annealing at 46 °C for 20 s, and elongation at 72 °C for 30 s. The quality of the amplification reaction was checked by generating a melting profile for each amplicon over a temperature range of 60–95 °C.

Each qPCR assay was performed using a Lightcycler^®^ 480 Instrument II (Roche, Indianapolis, IN, USA). A Cp value ≤39 was considered positive and ≥40–45 was considered negative. The limit of detection for each species-specific qPCR assay was 100 cells mL^−1^ (Supplementary Figure S1). In addition, the specificity and quality of positive amplification reactions were further confirmed by running the amplified product on a 2% agarose gel matrix (Fisher Scientific, Pittsburgh, PA, USA) in a 1× TAE buffer. The gels were stained with ethidium bromide (0.5 μg mL^−1^) and scanned with an AlphaImager UV transilluminator (Fisher Scientific, Pittsburgh, PA, USA).

### 2.4. Data Analysis

McNemar Chi-square Contingency and Fisher’s Exact tests were applied for assessing the rate of prevalence and comparing significant differences (*p* < 0.05) between *C. jejuni* and *C. coli,* and pre- vs. post-treatment data comparison in both communities was conducted using STATISTICA [32]. R [33] program was used for exploratory data analysis and to investigate the association between *C. jejuni* and *C. coli* with chlorine concentrations (free and total) in each sampling point.

## 3. Results

### 3.1. Prevalence and Cell Concentration of C. jejuni and C. coli

Overall, *C. jejuni* was detected at a relatively low frequency in all pre- and post-treatment samples, except SP. Of the 220 total samples, 26% (*n* = 57) of samples showed positive amplification for *C. jejuni*. Treated water in the WTP had a greater frequency of detection (*n* = 7; 37%) than S (*n* = 5; 26%) or T samples (*n* = 2; 11%). It was also commonly detected in samples from households with CP (*n* = 18; 31%) and CC (*n* = 8; 17%) and P (*n* = 15; 28%), as well as in both samples collected in the household with the CF (*n* = 2; 100%). Overall, there was no significant (*p* > 0.05) difference in the frequency of prevalence between pre- and post-treatment as well as among post-treatment samples. In addition, the lagoon (L) and septic tank (ST) reference sites showed positive amplification for *C. jejuni* at the frequency of 50% (*n* = 2) and 13% (*n* = 2), respectively.

In comparison to *C. jejuni*, a relatively higher frequency of *C. coli* was detected with an insignificant (*p* > 0.05) difference between pre- and post-treatment samples. Of the 220 total samples, 32% (*n* = 89) of samples were positive for *C. coli*. All CF and SP samples tested positive for *C. coli* as compared to S (*n* = 8; 42%), WTP (*n* = 8; 42%) and T (*n* = 9; 47%) samples. Similarly, *C. coli* was also frequently detected in tap water of households with CP (*n* = 25; 42%) and CC (*n* = 19; 41%) cisterns, as well as P (*n* = 16; 30%) samples (Table 2). However, no significant (*p* > 0.05) difference among post-treatment samples was observed. Of the total 37 (42%) positive samples for co-occurrence, *C. jejuni* and *C. coli* were often detected together in tap water of households with CP (*n* = 15; 41%) cisterns. Of the samples collected at other points in the distribution systems, households with P (*n* = 7; 19%) and CC (*n* = 4; 11%) cisterns, as well as the home with the CF (*n* = 2; 5%) cisterns, showed co-occurrence of *C. jejuni* and *C. coli.* In addition, S (*n* = 3; 8%), WTP (*n* = 5; 13%), and T (*n* = 1; 3%) samples also showed co-occurrences. Although both target species were detected at all sampling points in both communities at variable frequency, Community D showed a significantly (*p* < 0.05) high prevalence rate compared to Community B. Overall, *C. coli* was detected at a relatively high frequency (ranging from 18 to 100%) as compared to *C. jejuni* (ranging from 7 to 100%) (Table 3).

**Figure 2:**
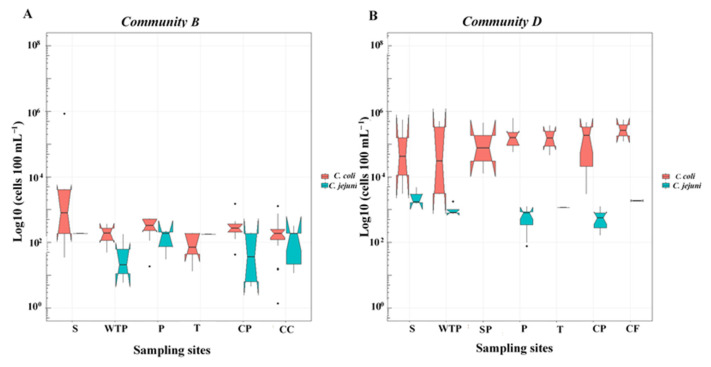
Concentration (cells 100 mL^−1^) of *C. jejuni* and *C. coli* in pre- and post-treatment samples collected from DWDS of two communities (panels **A** and **B**).

The cell concentration of *C. jejuni* was variable across all sampling sites ranging from 4.4 × 10 to 4.9 × 10^3^ cells 100 mL^−1^. The relatively high cell concentration (1000 cells 100 mL^−1^) was detected in 10 (18%) as compared to 100 cells 100 mL^−1^ in 36 (63%) samples. A substantial number of households with CP cisterns (*n* = 14; 39%) and P water (*n* = 11; 31%) had 100 cells 100 mL^−1^ cell concentrations. Similarly, cell concentrations in all three positive samples from the two reference (L and ST) sites had 100 cells 100 mL^−1^ of *C. jejuni.* In both communities, detection of 1000 cells 100 mL^−1^ cell concentrations occurred for *C. jejuni* at most sampling points, except in homes with CC cisterns with relatively low frequency. Except one T sample in Community B, the concentration of *C. coli* cells was always higher than *C. jejuni* (Figure 2). Notched box plots in Figure 2 were created to graphically compare the differences in the distributions of the concentrations of *C. coli* and *C. jejuni* cells in Communities B and D and in various sampling points. The number of samples of each box plot is provided in Table 3. In these boxes, the central line represents the median value. Instead of mean, the median was selected representing the central value since even after the logarithmic transformation the statistical distribution of the value was not completely normal and had some skewness as represented by the asymmetrical shape of the box plots. The interval of values represented by the notches of each box plot can be interpreted as a comparison interval around the median values. No overlap between the notches of two boxes can be considered as strong evidence that their medians are significantly different from each other at 95% confidence [34]. The notched box plot can be an alternative if the requirements of statistical hypothesis tests are not strictly met [34] as it is here by considering the limited number of samples in some sampling points (Table 3). The small sample size of the subsets in each sampling point (Table 3) causes the notches of boxes of Figure 2 to extend beyond the boundaries of the box (25th and 75th percentiles) due to the uncertainty of the true median value. As a result, the notches of the median can go beyond the hinges of the box and are displayed as folded inside (Figure 2).

### 3.2. Association between Campylobacter spp. and Chlorine

**Figure 3:**
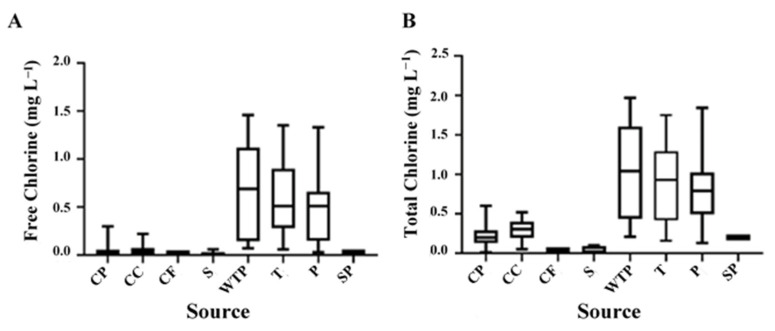
Concentration of free (panel **A**) and total (panel **B**) chlorine (mg L^−1^) in each source of drinking water sampling point.

**Figure 4:**
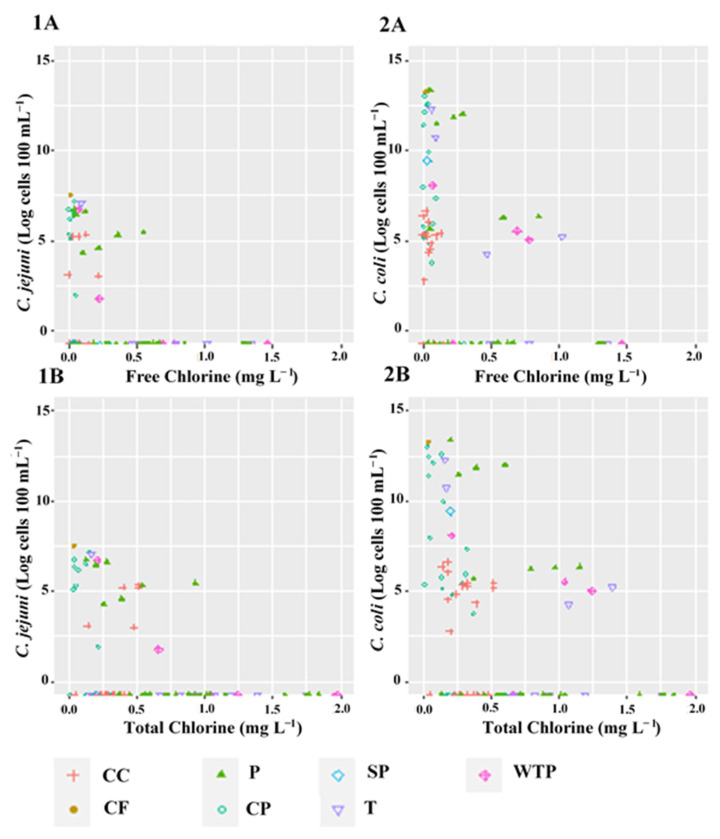
Correlation analysis between *Campylobacter* spp. and chlorine (free and total) across each sampling point: *C. jejuni* (panels: **1A** and **1B**) and *C. coli* (panels: **2A** and **2B**) vs. free and total chlorine.

The free and total chlorine in various drinking water sampling points ranged from 0.01 to 1.02 mg L^−1^ and 0.01 to 1.39 mg L^−1^, respectively. However, the free and total chlorine concentrations showed significantly (*p* < 0.05) higher average chlorine concentrations in WTP, T and P compared to CP, CC, CF, SP and S samples (Figure 3). As a step of the exploratory data analysis, scatterplots of the concentrations of *C. coli* and *C. jejuni* cells and free and total chlorine concentrations were created (Figure 4). As Figure 4 displays, no consistent linear, non-linear or clustered association can be identified to represent the association between the concentrations of *C. coli* and *C. jejuni* cells and free and total chlorine concentrations (Figure 4). The results showed that *C. jejuni* was detected at variable concentrations of free (ranged from 0.01 to 0.78 mg L^−1^) and total (ranging from 0.02 to 1.23 mg L^−1^) chlorine as compared to *C. coli* (free chlorine: 0.01–1.02 mg L^−1^; total chlorine: 0.01–1.39 mg L^−1^), respectively (Figure 4). This indicates that both target *Campylobacter* spp. were not inhibited by low levels of free and total chlorine.

## 4. Discussion

The poor microbiological quality of drinking water in many First Nations communities in Canada continues to be a serious problem. These concerns have led to the residents not trusting their tap water and having to spend a substantial amount on bottled water [29]. The households that do not have access to the piped water commonly use above- or underground cisterns for the storage of water. However, the cisterns are commonly underground to avoid effects of changes in surface temperature. The homes with underground cisterns are often equipped with an underground septic tank to collect domestic wastewater, which needs to be at least 8 m from the household cistern. The wastewater is trucked to a facultative lagoon system operated by the community. About one in every five homes in Community D rely on cisterns. In total, 6 in every 10 homes in the community are without water services. Although recent community developments are providing newly built homes with direct connections to the WTP. Currently, there are no CC cisterns in Community D and CP cisterns have been newly installed in this community as well. At least one household in Community D had a CF cistern which was included in this study. The CC cisterns are being preferably replaced with CP and CF cisterns as these materials are water- and rustproof. Moreover, they can easily be cleaned, disinfected and maintained. However, the quality of the water, stored in cisterns, may decline by the time it reaches the end-user, as demonstrated for detections of microbial water quality indicators in the tap water of homes in First Nations studies [9,35]. This may occur due to recontamination of water in pipes, trucks and cisterns after treatment by regrowth of injured or stressed bacteria or contamination from bacteria harbored in biofilms [36,37]. Moreover, variable chlorine concentration and stability may also be impacted by drinking water treatment processes, disinfection conditions and various distribution system properties [38]. Among several bacterial, viral and protozoal waterborne pathogens, campylobacters have been detected as a cause of several drinking-water-related outbreaks where the main risk for contamination was use of unchlorinated surface water and the secondary contamination of drinking water in storage reservoirs [1,39,40,41].

Therefore, we assessed pre- and post-treatment DWDS for the prevalence rate of thermophilic *Campylobacter* spp. (*C. jejuni* and *C. coli*) in two FN communities. Culturing of fastidious bacterial pathogens can often result in an underestimation of the bacterial load since they rapidly enter the viable but non-culturable (VBNC) state. Thermophilic *Campylobacter* spp., in particular, is one of the examples that form VBNC cells [42]. Due to the potential to cause risk of infection in humans, the VBNC cells present during water disinfection are important for drinking water safety and human health [43,44]. Therefore, detection and quantification of these bacterial pathogens by qPCR is preferred. In this study, we used species-specific probe and SYBR Green-based qPCR assays with high sensitivity for quantitative detection of total number of *C. jejuni* and *C. coli* cells [25,28,30] in pre- and post-treatment DWDS. Hence, our results demonstrate that the selection of a culture-independent method is important when testing for waterborne human pathogens in aquatic samples. Since the total number of each target species was quantified by using species-specific qPCR assays, so in the context of waterborne pathogen detection, total number of cells including viable and culturable, VBNC, and non-viable and non-culturable (NVNC) complicates the estimation of targeted pathogens in water by PCR [26,45,46]. The detection and quantification of NVNC cells may result in positive signals in qPCR analyses [47,48]. The residents that consumed drinking water positive for target *Campylobacter* spp. may have been exposed to risks of infection during the period prior to the sampling point even if the DNA, considering the rate of DNA degradation, that entered was from dead cells [47]. Further, qPCR-based positive samples would be important as an indication of recent or past contamination with these species. *Campylobacter* spp. has a low survival rate in the environment due to microaerophilic properties, the low infectious dose and VBNC state in adverse environmental conditions may affect the high prevalence of human campylobacteriosis attributed to water despite the low detection rate [45,49].

Overall, both target *Campylobacter* spp. were commonly prevalent in pre-treatment (S) (*n* = 13; 68%) and all post-treatment (*n* = 133; 66%) water samples. However, a low frequency of co-occurrence of both species in individual water samples was observed. While some contamination sources such as agricultural activities might be common in other areas, they do not apply in this instance since neither of these communities are agricultural communities. However, wildlife, particularly migratory birds, cannot be ruled out and may need further investigation [17,19,49,50,51]. The study results indicate that chlorination treatment may be operating efficiently against *Campylobacter* spp., but these bacteria may enter into the distribution system through various possible means including aging infrastructure including breaks, repairs and storage time in cisterns, pressure losses and related pressure losses that may lead to contamination [52]. Moreover, compliance monitoring of pipes that are directly connected from WTP to homes may also not identify short- or long-term contamination (e.g., polluted rainwater or faulty septic tanks) or treatment inefficiencies that can lead to cause waterborne gastrointestinal infection [53,54]. Therefore, the Point of Use filters [4] would help in reducing the risk of exposure to pathogens.

The study also assessed and compared the cell concentration of both *Campylobacter* spp. across all pre- and post-treatment sampling points in two communities using the qPCR method. The infectious dose of *C. jejuni* for humans from water is ~500 cells [55,56], where our results usually showed a similar concentration of *C. jejuni* and *C. coli* cells. However, occasionally the highest cell concentration (100,000 cells 100 mL^−1^) was observed in samples across all, except CC, sampling points as compared to 10,000 cells 100 mL^−1^ concentration in T, P and CP samples. Our results suggest that there is a potential risk of *Campylobacter* infection from the consumption of this water. Since we did not perform an assessment of potential human health risks associated to these samples, further research is required for such determination. All sampling points were detected with variable concentrations (ranged from 4.4 × 10 to 4.9 × 10^3^ cells 100 mL^−1^) of *C. jejuni* and *C. coli* (1.3 × 10 to 5.6 × 10^5^ cells 100 mL^−1^) which suggests that fecal wastes from lagoons, septic tanks and wildlife might be a source of contamination [18]. The high concentration of these two *Campylobacter* species by qPCR assays may possibly be due to the presence of VBNC and NVNC cells or free DNA [28].

Among fastidious bacterial species, it has been reported that VBNC cells of *Campylobacter* remain in water for an extended period of time ranging from weeks to months [43,57]. Moreover, *Campylobacter* has a poor recovery rate on selective media which indicates that it would substantially influence rate of recovery and detection from the drinking water distribution system if we applied an enrichment-culture-based method because the cells might be dead or injured by chlorine during the treatment process and the VBNC cells could not be recovered. Since we applied the qPCR assay that detected and quantified the total including viable and culturable, VBNC and NVNC number of cells that may have resulted in an overestimation of both target species in the pre- and post-treatment samples [28]. Although it has been suggested that the enrichment-PCR technique could be a preferable method for the enumeration of *Campylobacter* cells. However, this method has an inability to identify and enumerate VBNC and NVNC cells which would cause a serious problem in the detection and identification of the prevalence of pathogens that are present in a low concentration. Therefore, further research is being planned to accurately quantify viable and dead cells prior to the qPCR assay using propidium monoazide (PMA) or ethidium monoazide (EMA). However, the toxic effect and penetration of these dyes into viable or dead cells can either under- or over-estimate total number of cells [58,59,60]. Therefore, an optimization of dye concentration and exposure time may not impact the qPCR assay.

Although *C. jejuni* and *C. coli* have been identified as a major cause of bacterial gastroenteritis in Canada [61], one of the limitations of this data is that during the sampling time there were no cases of campylobacteriosis reported by public health that could be used for risk assessment analysis, source of contamination and *Campylobacter* infection. Since *Campylobacter* infection is self-limiting and does not require treatment, it is also possible that campylobacteriosis cases are under-reported. Similarly, the source of infection is difficult to identify due to the long incubation period of *Campylobacter* infections [62,63]. Further research is required to the assess seasonal impact on the prevalence and persistence and other physico-chemical parameters (e.g., pH, temperature, precipitation, etc.) that can be used for predicting occurrence or concentrations of *Campylobacter* spp. in pre- and post-treatment samples at each sampling point. In addition, microbial source tracking (MST) library-dependent or library-independent techniques are useful tools that can be applied to identify the sources of human and/or animal fecal pollution [64].

The free and total chlorine was significantly (*p* < 0.05) lower than the detectable concentrations that were below the acceptable Health Canada drinking water standards of free (0.2 mg L^−1^) and total (1.0 mg L^−1^) chlorine [4]. According to Manitoba’s provincial drinking water quality standards, the minimum allowable free chlorine at the point of water distribution system is 0.5 mg L^−1^ [65]. Our data indicate that 0.56 mg L^−1^ free and 0.93 mg L^−1^ total chlorine can reduce the number of *C. jejuni* in DWDS. However, 1.04 mg L^−1^ free and 1.35 mg L^−1^ total chlorine can reduce the number of *C. coli* in major distribution sources including WTP, T and P. Our results showed congruence with a previous study [66] where low (0.3 mg L^−1^) free chlorine concentration effectively reduced certain fraction of bacteria. The study also showed that the rate of samples containing viable bacteria exceeded 28% in 155 Beijing tap water samples where an incomplete removal of bacteria was recorded which indicates that survival and re-growth of bacteria in water distribution systems occur in the presence of chlorine at various concentrations.

## 5. Conclusions

This study provided data on the prevalence of human-associated thermophilic *Campylobacter* spp. and their association with free and total chlorine in pre- and post-treatment DWDS in two First Nation communities. The two target thermophilic *Campylobacter* (*C. jejuni* and *C. coli*) species were also detected in every drinking water sampling point where *C. jejuni* was predominantly detected in the CF cistern, WTP, and P compared to the S water samples. However, *C. coli* was more frequently detected in all pre- and post-treatment sampling points. The prevalence of both species indicated fecal contamination can potentially be from human, livestock and wildlife sources. While comparing the two First Nation communities, both *Campylobacter* spp. were detected in Community D at a significantly (*p* < 0.05) higher frequency than in Community B. This indicates that chlorination was not effective at removing *Campylobacter* spp. from the WTP as well as other factors such as breaks, repairs and storage time may potentially lead to contamination in various points of distribution system. The concentration of both *Campylobacter* spp. ranged from 4.4 × 10 to 4.9 × 10^5^ cells 100 mL^−1^ across all sampling points showing potential health risk to the First Nation communities. This data may provide useful information for human health monitoring to identify the sources of contamination. Since in this study no association between target *Campylobacter* spp. and chlorine was observed, therefore, there is an urgent need to directly measure the prevalence of waterborne human pathogens at each treated sampling point to better understand the treatment efficiency and patterns of prevalence and persistence. In order to identify the pathways of animal and human fecal pollution, further MST-based research is warranted that would help in developing remediation and quantitative microbial risk assessment strategies to improve drinking water quality.

## Figures and Tables

**Table 1:** Sample ID, source and site description of water samples, collected from pre- and post-treatment DWDS and fecal sources between July 2017 and October 2018, used in this study.

Sample ID	Source	Total Number of Samples	Site Description
S	Raw drinking source water	19	Surface water of the nearby lake drawn into the WTP as pre-treatment raw water from the WTP tap
WTP	Water treatment plant	19	Post-treatment water from the WTP tap
T	Water delivery trucks	19	Post-treatment water filled into potable water trucks transported homes with cisterns
P	Piped home	54	Home directly piped to the WTP
CP	Cistern (polyethylene)	59	Polyethylene cistern housed underneath the house or in an adjacent shed
CC	Cistern (concrete)	46	Exclusive to Community B; concrete cistern underground nearby but external to the home
CF	Cistern (fiberglass)	2	Exclusive to Community D; underground
SP	Standpipe	2	Exclusive to Community D; a 24 h community accessible standpipe directly piped from the WTP
L	Lagoon	4	Exclusive to Community B; grab sample from a two-cell facultative lagoon where septic delivery trucks transported wastewater from septic tanks
ST	Septic tank	16	Exclusive to Community B; grab sewage sample from septic tanks

**Table 2:** Total number (percent) of water samples positive for *C. jejuni* and *C. coli* in pre- and post-treatment DWDS of two First Nations communities.

Sampling Source	Total Number of Samples		*C. jejuni*	*C. coli*
	Community B	Community D		
S	15	04	5 (26)	8 (42)
WTP	15	04	7 (37)	8 (42)
T	15	04	2 (11)	9 (47)
P	40	14	15 (28)	16 (30)
CP	45	14	18 (31)	25 (42)
CC	46	0	8 (17)	19 (41)
CF	0	2	2 (100)	2 (100)
SP	0	2	0	2 (100)

**Table 3:** Total number (percent) of *C. jejuni* and *C. coli* positive samples collected from pre- and post-treatment DWDS.

Community B							Community D					
Sampling Source	S * *n* = 15	WTP *n* = 15	T *n* = 15	P *n* = 40	CP *n* = 45	CC *n* = 46	S *n* = 04	WTP *n* = 04	T *n* = 04	P *n* = 14	CP *n* = 14	CF *n* = 02
***C. jejuni***	2 (13)	3 (20)	1 (7)	3 (8)	12 (27)	8 (17)	3 (75)	4 (100)	1 (25)	12 (86)	14 (100)	2 (100)
***C. coli***	6 (40)	4 (27)	5 (33)	8 (20)	8 (18)	19 (41)	2 (50)	4 (100)	4 (100)	8 (57)	13 (93)	2 (100)

* = total number of water samples collected.

## Data Availability

The dataset presented in the current study is not publicly available since data contains information that is being further used for other publication; however, it can be available to the editor upon request.

## Supplementary Materials

The following supporting information can be downloaded at: https://www.mdpi.com/article/10.3390/ijerph191710466/s1, Figure S1: Development of standard curves for *C. coli* (Panel A) and *C. jejuni* (Panel B) generated based on amplification of DNA from increasing number of cells (A–F: 10^7^, 10^6^, 10^5^, 10^4^, 10^3^, and 10^2^ cells mL^−1^) using species-specific TaqMan probe and SYBR Green-based quantitative real-time PCR assays.
