# Supplementary material for: Quantitative Assessment of First Nations Drinking Water Distribution Systems for Detection and Prevalence of Thermophilic Campylobacter Species
Source: Int J Environ Res Public Health. 2022 Aug 23;19(17):10466. doi: 10.3390/ijerph191710466 (PMC9518054; doi:10.3390/ijerph191710466)
Supplement: Supplementary file 1 [file ijerph-19-10466-s001.zip › ijerph-1830968-supplementary.pdf]

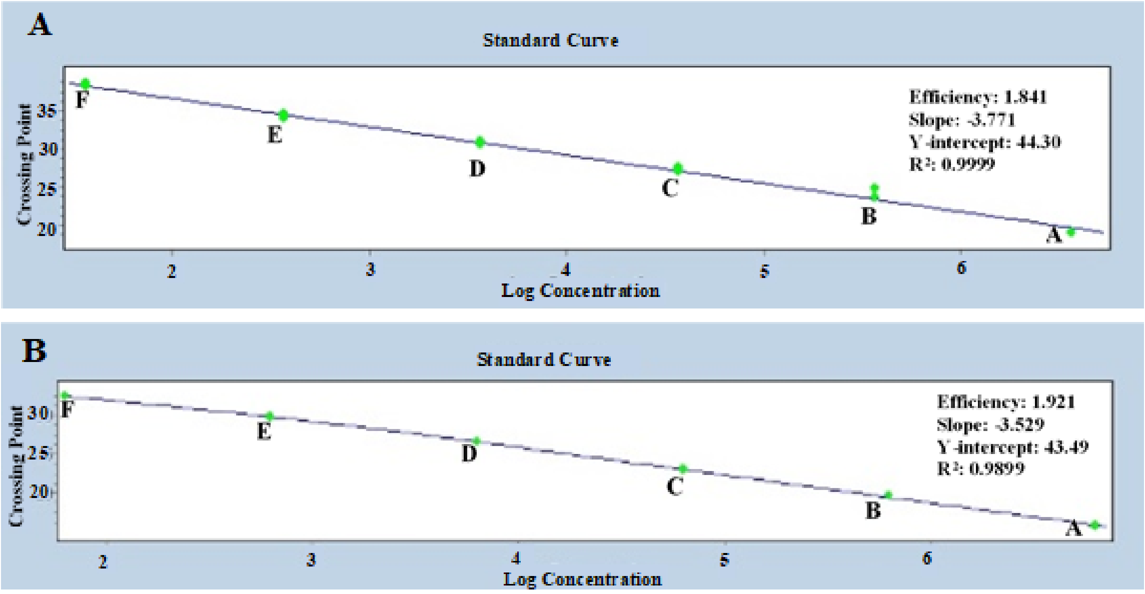

Figure S1: Development of standard curves for *C. coli* (Panel **A**) and *C. jejuni* (Panel **B**) generated based on amplification of DNA from increasing number of cells (A–F: 10^7, 10^6, 10^5, 10^4, 10^3, and 10^2 cells mL^{-1}) using species-specific TaqMan probe and SYBR Green-based quantitative real-time PCR assays.
